# A Web Service Protocol Realizing Interoperable Internet of Things Tasking Capability

**DOI:** 10.3390/s16091395

**Published:** 2016-08-31

**Authors:** Chih-Yuan Huang, Cheng-Hung Wu

**Affiliations:** 1 Center for Space and Remote Sensing Research, National Central University, Taoyuan 320, Taiwan; 2 Department of Civil Engineering, National Central University, Taoyuan 320, Taiwan; 103322089@cc.ncu.edu.tw

**Keywords:** Internet of Things, tasking capability, interoperability, OGC SensorThings API

## Abstract

The Internet of Things (IoT) is an infrastructure that interconnects uniquely-identifiable devices using the Internet. By interconnecting everyday appliances, various monitoring, and physical mashup applications can be constructed to improve human’s daily life. In general, IoT devices provide two main capabilities: sensing and tasking capabilities. While the sensing capability is similar to the World-Wide Sensor Web, this research focuses on the tasking capability. However, currently, IoT devices created by different manufacturers follow different proprietary protocols and are locked in many closed ecosystems. This heterogeneity issue impedes the interconnection between IoT devices and damages the potential of the IoT. To address this issue, this research aims at proposing an interoperable solution called tasking capability description that allows users to control different IoT devices using a uniform web service interface. This paper demonstrates the contribution of the proposed solution by interconnecting different IoT devices for different applications. In addition, the proposed solution is integrated with the OGC SensorThings API standard, which is a Web service standard defined for the IoT sensing capability. Consequently, the Extended SensorThings API can realize both IoT sensing and tasking capabilities in an integrated and interoperable manner.

## 1. Introduction

### 1.1. Background

The Internet of Things (IoT) is an infrastructure that interconnects uniquely-identifiable devices using the Internet. While the IoT is attracting attention from various fields, it is not a brand new concept. From the early stage of the 20th century, similar concepts were proposed, and some related communication technologies like the radio, barcodes, the Internet and radio frequency identification (RFID) were also invented [1,2]. At the early stage of the IoT, the main focus of IoT was to identify and track every physical thing, and many applications such as warehouse management and logistics applications applied RFID technology to prove the concept [1,3].

With the advances of communication and sensor technologies in recent years, the definition and scope of IoT have been extended. For instance, the International Telecommunication Union (ITU) defined the IoT as “a global infrastructure for the information society, enabling advanced services by interconnecting (physical and virtual) things based on existing and evolving interoperable information and communication technologies [4]”. ITU shows that every physical thing can have a virtual identity in the information world. Through those virtual identities, every thing can interconnect and communicate with each other.

With the extended definition and scope, the IoT is no longer confined to object identification applications. Everyday appliances, such as TVs, ovens, heaters, lamps, door locks, could be connected to the Internet via different local communication technologies (e.g., Bluetooth and Zigbee, WiFi). By connecting devices to the Internet, users and applications can access device capabilities in a remote and real-time manner. Hence, the vision of the IoT started to attract the attention of various domains and applications. For example, Gubbi et al. [5] mentioned that IoT applications have four main categories: (1) Personal and Home; (2) Enterprise; (3) Utilities; and (4) Mobile. Atzori et al. [2] think that the IoT can be applied in: (1) Transportation and Logistics; (2) Healthcare; (3) Smart environment (home, office, plant); (4) Personal and social. Meanwhile, many predictions estimated that the number of IoT devices would reach 20 billion to 50 billion by 2020 (http://www.gartner.com/newsroom/id/3165317). As we can see from these predictions and envisioned applications, the IoT is one of the most popular developments in recent years. 

In general, the IoT has two main capabilities, which are sensing and tasking capabilities:

(1). Sensing capability
  The sensing capability monitors devices’ statuses or the environmental properties of their surroundings. Users can remotely capture the properties for data analysis and application. People can use different sensors to collect not only the environmental properties like temperature, humidity, and location information, but also the status of devices such as “On” or “Off”. While sensors can monitor various properties, sensor owners or users can remotely access the sensor observations through the Internet. For example, users can understand if a device is “On”, or the battery status of each device. In general, the sensing capability allows users to remotely monitor device statuses and various properties, and consequently, users can utilize the sensor observations to support automatic and efficient applications.
(2). Tasking capability
  The tasking capability allows other devices or users to actuate devices via the Internet so the users can easily control the devices to execute feasible tasks remotely. For example, existing IoT products such as Philips Hue and Belkin WeMo provide tasking capabilities. The Philips Hue allows users to remotely turn on or off light bulbs as well as adjust brightness and saturation via the Internet. The Belkin WeMo is a smart switch. Users can connect their traditional appliances to the WeMo and remotely turn on or off these appliances by controlling the WeMo. Overall, while the sensing capability allows users to continuously monitor the statuses of devices and the environmental properties, the tasking capability can help users to make adjustments accordingly by controlling devices remotely.

In general, *mashing-up* the sensing and tasking capabilities of different IoT devices enables users to create various automatic and efficient applications. These kinds of applications are called “physical mashup” applications [6]. For instance, an application can use the GPS sensor in a mobile phone to monitor a user location. When the application senses that the user is heading back home, the application can automatically turn on air conditioners or heaters. In this case, the house will be at a comfortable temperature when the user arrives home. As a result, to facilitate the mashups of IoT capabilities, one of the key requirements is to provide a uniform interface for users or applications to access the capabilities in an interoperable manner.

### 1.2. The IoT Architecture

**Figure 1:**
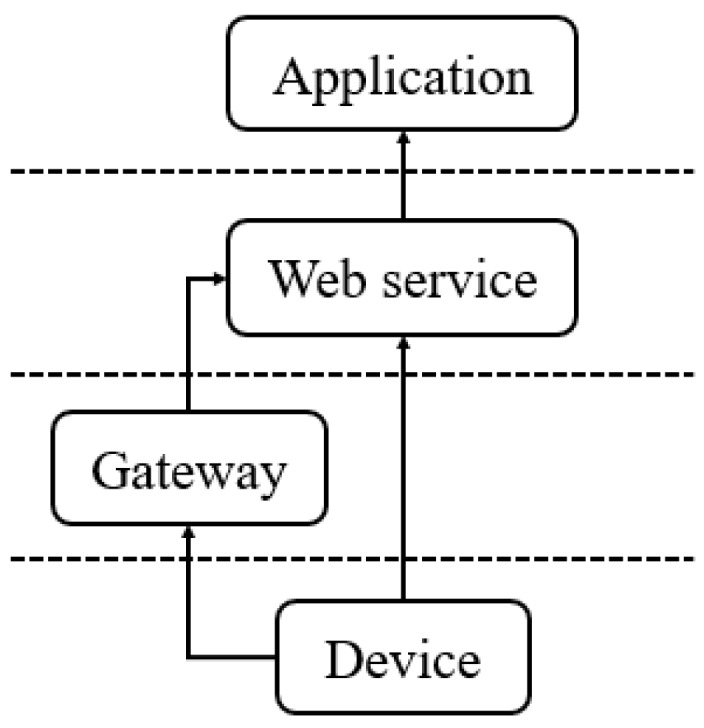
Architecture of Internet of Things.

To further define the scope of this research, here we introduce the IoT architecture. As shown in Figure 1, there are four layers in the architecture which are Device Layer, Gateway Layer, Web service Layer and Application Layer [2]. The Device Layer contains the devices connecting to the Internet, such as appliances and smart sensors. With the ability to connect to the Internet, devices can upload sensor observations to a web service or be controlled by users via the Internet. However, devices can be divided into two types. The first type of device has enough computation resource to directly connect to the Internet. The second type of device is the device that is too resource-constrained to directly connect to the Internet by itself.

Therefore, an additional layer called the Gateway Layer is required to serve as an intermediate layer to connect the resource-constrained devices on one end and connect to the Internet on the other end [7]. Usually, gateways act as a translator converting the device protocol to the web service protocol and vice versa. After connecting to the gateways, the resource-constrained devices will be able to connect to the Web Service Layer.

The Web Service Layer contains services that may receive data from gateways or directly from devices. Different services may provide different functionalities, such as data processing, data storing, data management and query. Finally, the Application Layer is where applications retrieve the resources from the Web services and usually provide graphical user interfaces for users to operate and consume the IoT data.

In order to achieve an interoperable IoT infrastructure, standards are necessary for the communications between layers. When two layers follow the same standard, they can communicate and understand each other based on the defined data model and protocol. In this case, the IoT can achieve interoperability [2,5]. With the increasing amount of Internet of Things devices, different and various IoT devices would cause heterogeneity issues. These heterogeneity issues could be solved by following certain standards to achieve the IoT interoperability [5,8].

In principle, two layers following the same standard can communicate with each other. While there have been some open standards proposed to support communication in the Gateway and Device layers, e.g., Bluetooth Low Energy, Zigbee, 6LoWPAN, and WIFI, we argue that a comprehensive IoT Web service standard is currently missing. Hence, this research mainly focuses on the communication between the Web Service layer and Internet-connected devices (including the resource-constrained devices connected to gateways).

### 1.3. Problems and Objectives

While the IoT is attracting much attention, companies are defining different proprietary web service protocols, and only their own IoT devices support those protocols. Either with the intention or without, these companies construct closed ecosystems, which we call the IoT silos. Each silo may have its complete IoT architecture and components including devices, gateways, services, and applications. However, the components in one silo cannot connect to the components in another silo as they support different communication protocols. For example, users usually need to use specific software or applications provided by different companies to access different IoT devices, such as the Philips Hue or the Belkin WeMo Switch, as these devices use different communication protocols. Hence, the IoT is currently suffering from a lack of interoperability and faces heterogeneity problem. This heterogeneity problem seriously damages the development of the IoT as users and applications cannot communicate with every IoT devices in a uniform way [5,8]. Moreover, the heterogeneity problem impedes the design of a unifying framework and the communication protocol [9]. As a result, since IoT devices cannot be easily connected, extra costs are required to achieve automatic and efficient physical mashup applications.

We identify two main approaches to address this IoT heterogeneity issue. The first one is the hub approach. The hub approach mainly focuses on developing different connectors to communicate with different IoT devices and provides users/applications a uniform interface, such as the Apple HomeKit, Alphabet (Google) Nest ecosystem and If-This-Then-That (IFTTT). The connectors could be in the Gateway Layer, Web Service Layer, or Application Layer. However, as the devices or services in different IoT silos support different protocols, the hub approach needs to implement every kind of connector to communicate with all the IoT silos, which would result in a large development cost.

The other approach is the open standard approach, which is usually the best approach to ultimately address heterogeneity issues [5,8]. There have also been some IoT standards proposed to address the IoT heterogeneity issue. Among which, the SensorThings API v1 standard [10] defined by the Open Geospatial Consortium (OGC). SensorThings API defines comprehensive query functionalities based on the OGC Observation & Measurements data model standard [11]. The SensorThings API provides a uniform service interface to host the virtual identities of IoT devices as well as the generated sensor observations. However, the IoT tasking capability is not supported by the first version of the SensorThings API, which means users/applications still cannot send requests to IoT devices with a uniform interface.

Therefore, to fill the gap, the goal of this study is to propose a potential solution to realize the IoT tasking capability in an interoperable manner. While a naïve solution is to define an IoT device protocol for manufacturers to follow, from a previous study experience, we can understand that manufacturers prefer having the flexibility to define their proprietary protocols instead of following a defined standard [12]. Therefore, we choose to define a web service protocol and a description data model to communicate with different IoT devices. Overall, this study has three main objectives:

(1). Firstly, this research aims at defining a common and uniform description document standard that can describe the communication protocols of IoT devices. This description allows the web service layer to automatically understand the device protocols. Currently, we only focus on HTTP protocols.
(2). Secondly, we propose a web service protocol that can automatically translate between users’ tasking requests and device protocol requests. Therefore, users/applications can use a uniform interface to control different IoT devices even if the devices follow different proprietary protocols.
(3). Thirdly, this research aims at integrating the proposed solution with the OGC SensorThings API, which supports the IoT sensing capability [10]. The integrated web service protocol is named the *Extended SensorThings API*. In this case, the Extended SensorThings API could be the solution that manages the virtual identities of IoT devices and supports both IoT sensing and tasking capabilities in an interoperable manner.

In the next section, this paper reviews the existing solutions for solving the heterogeneity problem and then indicates the limitations of these solutions. In Section 3, we first introduce the overall architecture of the Extended SensorThings API, as well as define the scope of this research, and then explain the proposed solution. In Section 4, we apply the proposed solution on some existing IoT products and demonstrate the contributions by constructing some applications. Finally, this paper concludes in Section 5.

## 2. Related Work

Interoperability of IoT implementations is a critical issue to be solved to realize the IoT vision. After reviewing the existing approaches addressing this issue, we categorize them into two approaches, which are the hub approach and the open standard approach.

The hub approach is an intuitive and common solution to solve the heterogeneity problem. A hub could connect with different IoT products by developing connectors following different communication protocols. Hubs could be in Gateway Layer, Web Service Layer, or Application Layer. Through the hubs, users can communicate with different IoT products. The hub approach can be effective, and many industrial companies have applied this approach to interconnect IoT devices, such as the Apple HomeKit, Alphabet (Google) Nest ecosystem and IFTTT. The key of these hubs is to develop connectors to support different IoT device communication protocols. For example, IFTTT is a web platform supporting many connectors for various IoT products. Users can use IFTTT “recipes” to connect their IoT devices and online services (e.g., email, calendar) to realize automatic applications. However, although the hub approach is effective, connectors specifically developed for different IoT products is necessary. As the number of IoT products increases rapidly, the cost of developing every possible connector get higher. Therefore, we believe that the hub approach is not the ultimate solution for the IoT heterogeneity issue.

On the other hand, the open standard approach defines standards to address the heterogeneity problem. In principle, with open standards, manufacturers and users can follow the same data model and communication protocol to create devices, gateways, web services and applications that can automatically understand each other.

There have been some efforts defining IoT standards. For example, the HyperCat is a standard proposed by Flexeye and other companies [13]. HyperCat provides a hypermedia catalogue standard to index and shares IoT resources (e.g., products and data) online [13]. As a catalogue service standard, HyperCat mainly focuses on the resource discovery functionalities and the heterogeneity problem of connecting IoT devices is not the focus. In addition, the OpenIoT and OM2M (https://wiki.eclipse.org/OM2M/one) standards both define web service protocols to manage IoT products and data [14]. However, these two standards still need to build different connectors to communicate with different IoT devices. Furthermore, the OGC Sensor Web Enablement (SWE) standards such as the Sensor Observation Service (SOS) [15] and Sensor Planning Service (SPS) [16] are also relevant. The SOS focuses on the sensing capability and defines service interfaces to help users to share sensor observations and sensor metadata. The SPS focuses on the tasking capability and defines service interfaces to help users control their sensors. However, as the SPS and aforementioned standards mainly defines web service interfaces, connectors should be implemented to control devices. Therefore, similar to the hub approach, these existing standards still cannot solve the heterogeneity problem of connecting IoT devices.

Although the intuitive approach to connect IoT devices is to define a uniform device communication protocol, previous experience tells us that manufacturers prefer having the flexibility to define their proprietary protocols instead of following a defined standard [12]. Therefore, we need a solution that allows users to communicate with IoT devices with a uniform interface while manufacturers can design their device protocols. In order to achieve this objective, we first consider the interaction between IoT devices and users. The interaction between users and IoT devices is similar to the interaction between client and server, in which, an IoT device can be regarded as a server that receives requests from clients. In tradition, before a client sends a request, the server needs a way to advertise its protocol. A web service description is a document describing the functionalities and communication protocols of the service [17]. With the web service description, a client could automatically understand how to communicate with the service.

There have been some existing web service descriptions such as the Web Service Descriptions Language (WSDL) [17] and the HTML Microformat for Describing RESTful Web Services (hRESTS) [18]. WSDL is an XML-based service description standard, and it defines not only the functionalities and protocols of services but also the format and schema of data. In recent years, while RESTful web services become popular and the WSDL is not suitable to describe RESTful web services, Kopecky et al. [18] proposed the hRESTS based on XHTML format to describe RESTful service protocols. However, even with hRESTS, users still need to develop connectors to communicate with different IoT devices [19].

**Figure 2:**
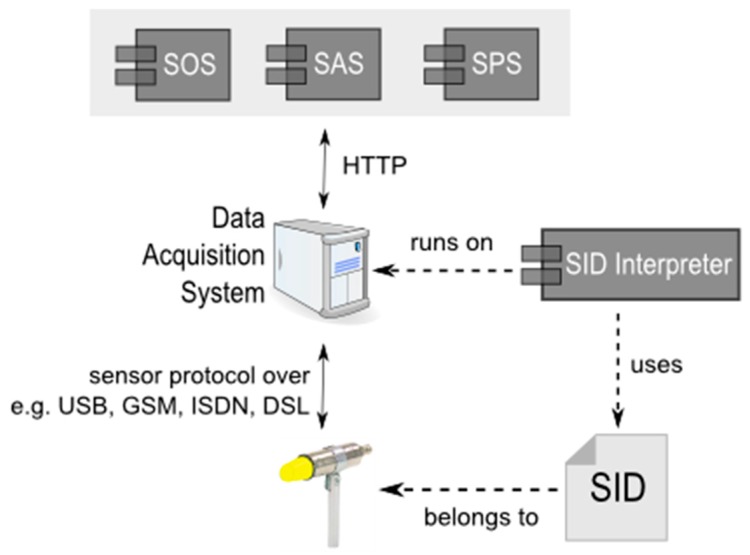
The architecture of SID [20].

A similar work to our proposed solution is the Sensor Interface Descriptor (SID), which is a declarative model based on the OGC Sensor Model Language (Sensor ML) standard for describing device capabilities [20]. As shown in Figure 2, the SID describes sensor metadata, sensor commands and device protocols. With a SID Interpreter, a data acquisition system can retrieve data from sensors and communicate with the SOS and SPS services [20]. SID provides a uniform interface for SWE standards and establishes the connection between the SWE web service standards and the sensors. In terms of the tasking capability, although SID seems able to describe device protocols with the OSI model (Open Systems Interconnection model), clear description and examples of how to automatically compose device requests based on SID seems to be missing from the references [20]. In addition, while SID presents a complete but complicated data model and verbose XML schema, to understand and adapt the SID may be costly for IoT device manufacturers. Therefore, this research proposes an alternative solution that uses a relatively simple data model to describe IoT device protocols.

In general, we argue that the existing solutions cannot fully address the IoT heterogeneity issue. This research focuses on developing a uniform service description for the IoT tasking capabilities that is flexible enough to describe different HTTP protocols for controlling IoT devices. Also, while most of the existing service description solutions are based on the XML format, the documents are usually large in size [21] and not suitable for resource-constrained IoT devices. Hence, the web service description this research proposes is based on the JSON format. Overall, by describing device protocols in a uniform manner, a web service can be constructed to connect automatically to different IoT devices, and consequently, users can control different devices using a single service protocol.

## 3. Methodology

This study aims to define an interoperable solution for the IoT tasking capability. The proposed solution has two main parts. Firstly, we define a JSON-based web service description to describe device tasking capabilities including the metadata and communication protocols. Secondly, for users to remotely connect with different IoT devices with a uniform interface, we propose a web service that can understand the proposed service description and automatically transform users’ tasks into device requests. We also specifically design the web service as an extension of the OGC SensorThings API based on the idea that the Extended SensorThings API could provide access to both IoT sensing and tasking capabilities. In this section, we first introduce the overall workflow of the proposed solution and the connection with the SensorThings API. Then we present the details of the proposed tasking capability service description. Finally, we explain the proposed solution with an example.

### 3.1. The Overall Workflow of the Extended SensorThings API

**Figure 3:**
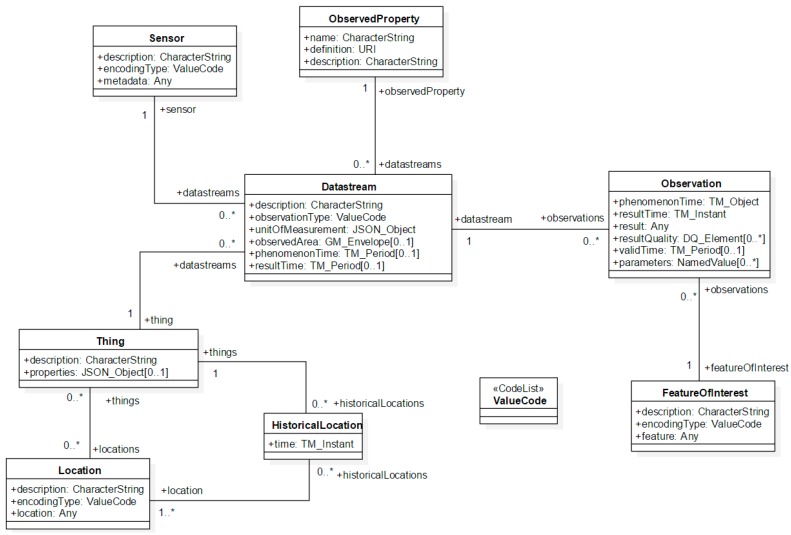
The data model of the OGC SensorThings API [10].

To propose a complete and interoperable solution for the IoT, this study integrates the proposed solution with the OGC SensorThings API [10]. The OGC SensorThings API provides an open and flexible web service protocol to host “things” and manage observations [10]. This standard is based on the JSON format and mainly follows the OGC Observation and Measurement (OGC O&M) model as the sensor observation model (Figure 3) [11]. In general, the first and current version of the SensorThings API is mainly designed for the IoT sensing capability and did not include the tasking capability. Therefore, to provide a complete IoT web service, this research proposes an interoperable solution as an extension to the SensorThings API, where the general operations (e.g., the Create, Read, Update, and Delete of entities) directly follow the SensorThings API standard. For the remainder of this paper, this extended service is referred to as Extended SensorThings API.

**Figure 4:**
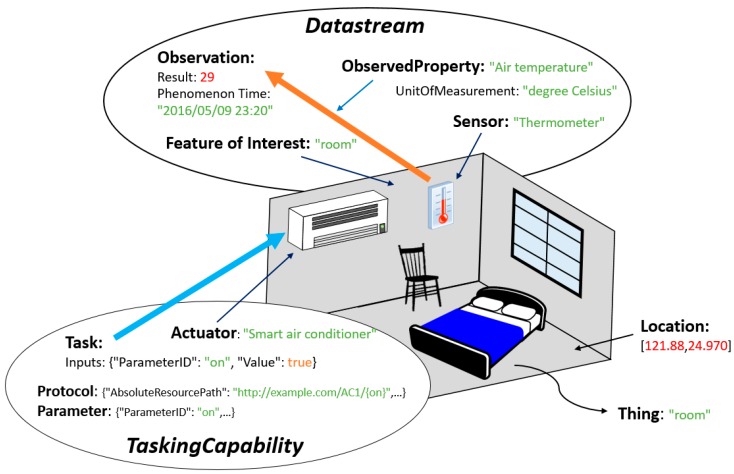
An example of the Extended SensorThings API.

While the details of the proposed tasking capability data model are presented in Section 3.2, here we use an example to explain the conceptual model of the Extended SensorThings API. As shown in Figure 4, regarding the sensing capability, a “room” can be regarded as a *Thing*, and the *Location* records the position information of the room. In this room, a “thermometer” is modeled as the *Sensor* measuring “air temperature” (i.e., *ObservedProperty*) of the room (i.e., *FeatureOfInterest*) with the *UnitOfMeasurement* “degree Celsius”*.* In this example, the sensor performs an *Observation* at *PhenomenonTime* 9 May 2016 23:20 and receives a *Result* of 29. This information is modeled as a *Datastream* of the *Thing*.

Regarding the tasking capability, this room has a “smart air conditioner” which could be described as an *Actuator*, which provides an ability for users to turn it on or off via HTTP requests*.* This ability is modeled as a *TaskingCapability* of the room (i.e., *Thing*) as an ability to adjust the temperature of the room. This *TaskingCapability* describes the acceptable parameters in *Parameters* and uses *HTTPProtocol* to record the device protocol template. With this information, users can create a *Task* to remotely control this *TaskingCapability* via the Internet. For example, to cool down the room, a user can set an *Input* with the parameter “on” as *true* in a *Task*.

One thing to note is that similar to the SensorThings API data model, users/providers have the flexibility to model their scenario. For example, a user could also model the air conditioner as a *Thing* instead of an *Actuator* providing *TaskingCapability* for the room. This decision is left for users/applications to decide. In general, the Extended SensorThings API can handle both sensing and tasking capabilities so that users can access complete IoT capabilities in a single service and integrate the capabilities for different applications. For example, an environment control application can retrieve temperature readings from Extended SensorThings API periodically. When the temperature exceeds predefined maximum or minimum thresholds, the application will automatically turn on or off the air conditioner by creating a *Task* entity.

After introducing the overall high-level model of Extended SensorThings API, one of the main objectives of this study is to design an IoT tasking capability solution so that users can control IoT devices with a uniform interface while allowing manufacturers to define proprietary device protocols. Before presenting the details, we first describe the key ideas and the overall workflow:

(1). Describe the IoT device tasking capability: To connect IoT devices while allowing manufacturers to define different protocols, this study proposes a uniform web service description format (presented in Section 3.2). By following the service description, users or manufacturers can first write documents to describe the tasking capabilities of IoT devices, which include the templates of device protocols.
(2). Register the IoT tasking capability to an Extended SensorThings API service: After preparing the tasking capability description, users, manufacturers or devices themselves can register the descriptions to a web service following the Extended SensorThings API. The web service can then automatically understand the device protocols of different IoT devices.
(3). Retrieve the tasking capability from the Extended SensorThings API service: Before users or applications control the IoT devices, they can retrieve the available tasking capabilities from the Extended SensorThings API to understand the allowed input parameters. While the SensorThings API provides a simple and RESTful interface for the user to retrieve resources, the Extended SensorThings API follows the same procedure. Based on the information in the tasking capability document users retrieved, users can create valid *task* entities (presented in Section 3.2) for controlling different tasking capabilities.
(4). Control IoT devices via the Extended SensorThings API service: Finally, users create *task* entities in the Extended SensorThings API service. The Extended SensorThings API will parse the input parameters and values, retrieve the corresponding tasking capability description from the database, and automatically compose a device request based on the protocol template in the tasking capability description. Finally, the service sends the request to control the IoT devices.

### 3.2. The Data Model of the Tasking Capability Description

**Figure 5:**
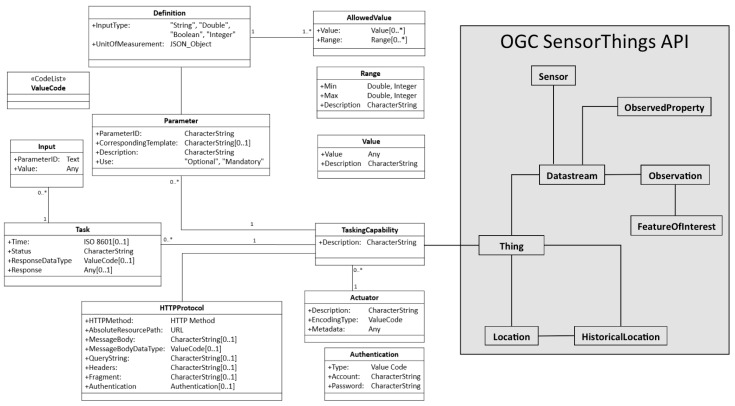
The data model of tasking capability.

As we mentioned earlier, IoT devices and products are similar to web services, which should have documents to advertise their capabilities and communication protocols. While XML format may not be suitable for resource-constraint IoT devices, this research tries to propose a JSON-based tasking capability document standard, which is called “tasking capability description”. In general, the tasking capability description is a JSON-based description that mainly describes the IoT tasking capability including the communication protocols and metadata of IoT devices. Figure 5 shows the data model of the tasking capability. To connect the proposed tasking capability with the SensorThings API, we can simply connect the *Thing* entity from the SensorThings API with the *TaskingCapability* entity, which represents the controllable ability of the *Thing*.

While the main purpose of the tasking capability description is to describe the protocol of IoT devices, a *TaskingCapability* entity uses the *HTTPProtocol* and zero-to-many *Parameter* entities to describe the protocol. The *HTTPProtocol* presents the template of the device request, which includes every component of an HTTP request. The *Parameter* records not only the description of *Parameter* but also the *Definition*, which defines the data type, unit of measurement, and *AllowedValues* so that users can understand the feasibly allowed values to the specific *Parameter*. In this research, we mainly focus on the HTTP-based protocol as most IoT devices are connected to the web. *Tasks* are the entities users send to the service to control IoT devices, which contain users’ input values in the *Inputs*. In Section 3.2.1, this paper introduces the details of each class.

For addressing the heterogeneity issue the IoT devices facing, the proposed tasking capability data model should be able to describe possible IoT device protocols. In this section, we introduce the details of each defined class.

#### 3.2.1. TaskingCapability

This research designs the *TaskingCapability* to be the controllable ability of a *Thing*. For example, a *Thing* can be an Internet smart light bulb, a smart lock or a smart plug. A smart light bulb could have tasking capabilities that can change its color, brightness, and saturation. In terms of the *TaskingCapability* class in the data model, *TaskingCapability* provides a uniform way to describe these controllable abilities of *Thing* so that users can understand the controllable abilities of the *Thing*. Moreover, one *Thing* can have multiple *TaskingCapabilities* to describe different controllable abilities. Table 1 shows that *Tasking Capability* only has one Description property to explain what this *TaskingCapability* is. The detailed information of the *TaskingCapability* is shown in the linked *Parameter*, *HTTPProtocol*, and *Actuator* entities.

#### 3.2.2. Actuator

The *Actuator* is used to describe the specific actuator of a *TaskingCapability*. For example, an Internet camera (as a *Thing*) may have a motor actuator, which can support two tasking capabilities. One is for the pan and tilt movement. Another is for the zoom in and zoom out. So an *Actuator* may link to multiple *TaskingCapabilities*. As shown in Table 2, *Actuator* has three properties. Description helps users understand the actuator. Metadata describes the detail of this actuator, which is usually based on a structured format. EncodingType presents the DataType of the Metadata. With the EncodingType, manufacturers can describe the Metadata of their IoT products in a standard format, e.g., the OGC SensorML, or in any data types such as plain-text, XML, and JSON.

#### 3.2.3. Parameter

The *Parameter* mainly has four properties (Table 3) for describing all of the acceptable parameters of the *TaskingCapability*. ParameterID shows the unique identification of an allowed parameter. Description can be used to describe the meaning of the parameter so that user could understand the use of it. Use is used to identify whether the parameter is “Optional” or “Mandatory”. CorrespondingTemplate records the corresponding portion of the template in the *HTTPProtocol* of a specific optional parameter. CorrespondingTemplate helps remove the unrequired portion from the template when composing a device request (details presented in Section 3.3). Also, the *Parameter* has a set of properties to describe the details, including the *Definition*, *AllowedValues*, *Value* and *Range* shown in Table 4, Table 5, Table 6 and Table 7, respectively.

One *Parameter* has one *Definition* that is used to describe the input data type and unit of measurement. Then one *Definition* may have multiple *AllowedValues* because a *Parameter* can have more than one acceptable value. For example, a smart light bulb may have a parameter “status”, and the allowed value of the “status” could be “On” or “Off”. Moreover, each allowed value should have one Description to describe the meanings of allowed values.

Also, while allowed values could be in a specific range, our proposed solution defines the *Range* class for this scenario. *Range* records Max, Min. For example, because the brightness of the smart light bulb may have a range (e.g., from 1 to 254), the *Range* can represent the maximum and minimum values of the brightness. In general, through these properties, the acceptable parameters of a *TaskingCapability* can be modeled completely.

#### 3.2.4. HTTPProtocol

In order to automatically communicate with different IoT devices, this research designs a class to describe possible communication protocols of IoT devices. In this study, we mainly focus on HTTP-based protocol [22]. While manufacturers could design device protocols in different ways, such as using different HTTP methods (e.g., GET, POST, PUT, DELETE) and putting input values in the URL, in HTTP headers, or in message body, in order to describe every possible protocol, we design the *HTTPProtocol* as a request template according to the device communication protocol.

In the template, we use the ParameterIDs in the *Parameters* as placeholders to indicate the location that input values should be. By simply replacing the placeholders in the template with input values, the Extended SensorThings API can automatically compose a request. We call this process “keyword replacement” and further explain it in Section 3.3. Therefore, to accommodate any possible device protocol, the *HTTPProtocol* contains properties for describing the common HTTP request components [22] as shown in Table 8.

In addition, while the privacy and security requirements are critical for some IoT applications, some IoT products/services require users to provide authentication information (e.g., username and password) when connecting to IoT devices. Therefore, the proposed *HTTPProtocol* and web service implementation can describe and support authentication standards such as the HTTP Basic Authentication and Digest Authentication [23]. Users can first register their authentication information with the *Authentication* properties shown in Table 9, and then the web service will automatically apply the specified authentication standard when sending device requests.

Through the above four classes in the tasking capability data model, the data model can completely describe the IoT device communication protocol. However, for users to control IoT devices in a uniform manner while following the general SensorThings API operation, this study designs the *Task* class as an entity type in the Extended SensorThings API.

#### 3.2.5. Task

While users can create entities in a general SensorThings API service by sending HTTP POST requests, we design our system so that users can send tasks by simply creating *Task* entities in an Extended SensorThings API service. In this case, users can control IoT devices in a uniform way. To be specific, the *Task* contains properties for a user to identify the *TaskingCapability* he/she wants to control, the time point for executing the *Task* (i.e., Time), and the input values for the Task (i.e., Inputs). For each Input, users should specify a ParameterID and a Value so that the Extended SensorThings API can use the ParameterID to find the placeholder in the *HTTPProtocol* template and replace the placeholder with the input Value. In addition, users can follow the SensorThings API Read operation to retrieve the created *Task* entities and find out their statuses (i.e., Status) and Responses from devices. The following Table 10 and Table 11 present the properties of *Task* and *Input*, respectively.

### 3.3. Keyword Replacement

To support any possible protocols that IoT devices could use, this study proposes an approach called “keyword replacement”. When users or manufacturers create a tasking capability description, the *HTTPProtocol* entity mainly presents the request template of device protocol. In the *HTTPProtocol* properties, manufacturers can place “keywords” at the locations where users’ input value should be. Then by simply replace the keywords with the Values in a Task, the service can automatically compose a device request. The designed style of the keyword is simple, which is using curly brackets (i.e., “{” and “}”) to encompass a ParameterID. For example, if a ParameterID is “On”, its keyword is “{On}”. We expect this approach could accommodate the heterogeneities in IoT device protocols and provide a uniform web service interface for users to access the IoT tasking capability.

**Figure 6:**
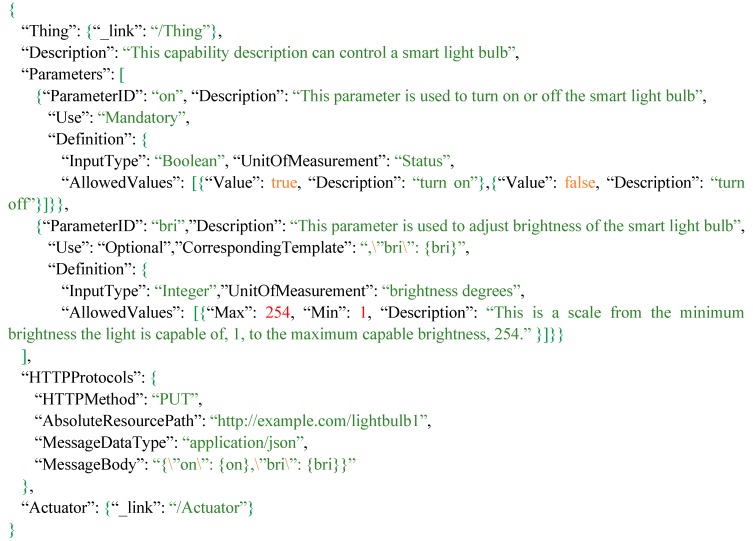
An example of a tasking capability description for a smart light bulb.

**Figure 7:**
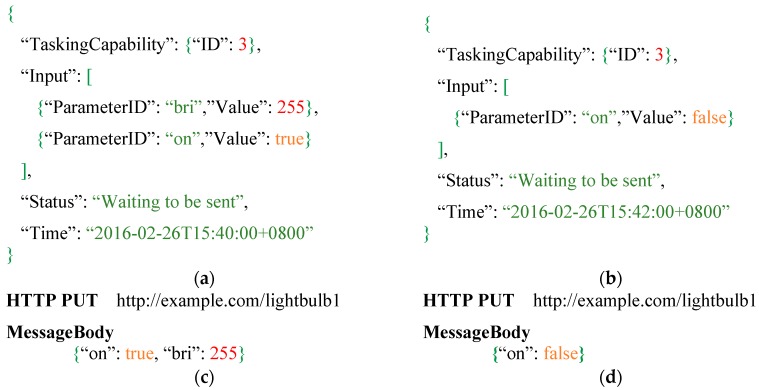
(**a**) A *Task* with two input parameters; (**b**) A *Task* with one input parameters; (**c**) The device request corresponds to the two-input-parameters *Task*; (**d**) The device request corresponds to the one-input-parameter *Task*.

Here we use an example to explain the keyword replacement process, for a scenario that a user owns a smart light bulb, he/she registered the tasking capability of the light bulb (Figure 6) to an Extended SensorThings API service. When this user sends a *Task* with the *Inputs* shown in Figure 7a to turn on the light bulb, the service will first parse the Inputs. The service will find the ParameterID “on” and “bri” with Value *true* and 255, respectively. Then the service composes keywords “{on}” and “{bri}” to find the placeholders in the *HTTPProtocol* template. In this case, the service will use the Value *true* and 255 to replace the “{on}” and “{bri}” in the MessageBody. As a result, the final device request is shown in Figure 7c.

In addition, while some *Parameters* are optional, and the *HTTPProtocol* records a complete template, the portion for those optional parameters should be removed from the final request if unrequired. For example, when users only want to turn off the light bulb by specifying the ParameterID “on” and Value *false* in the *Task* as shown in Figure 7b, the portion of the template for ParameterID “bri” is not required in this case. Hence, we design the CorrespondingTemplate in *Parameter* shown in Figure 6 to specify the corresponding portion of the template in *HTTPProtocol*. In this case, when the service is composing a device request, Figure 7d shows the service removes the CorrespondingTemplates of optional *Parameters* that are not required and composes a device request. Finally, if a request has a MessageBody, the service will use the MessageBodyDataType to verify and fix simple formatting issues in the MessageBody, such as removing necessary commas from a JSON String. As a result*,* the service can automatically compose a complete HTTP request for controlling an IoT device.

The above example shows the case of having input values in the MessageBody. However, with the defined tasking capability description and the keyword replacement procedure, input values can be automatically placed in other HTTP components (i.e., resource path, querystring, headers, or fragment). Users or manufacturers only need to put the placeholder in the correct place in the template, and the service can automatically locate the placeholders and compose a complete HTTP request. This solution is intuitive and simple enough to accommodate various device protocols and could consequently realize the interoperability of IoT tasking capability.

## 4. Results

The main focus of this study is to propose a uniform service description for describing heterogeneous IoT device protocols and design a web service interface for users to control different IoT devices following a single protocol. To demonstrate the contribution of this study, we first apply the proposed tasking capability description on three existing IoT devices, which are the Philips Hue, Belkin WeMo Switch, and a Panasonic IP camera. Secondly, we use the Extended SensorThings API to create two physical mashup applications to demonstrate the potential usage of the proposed solution.

### 4.1. The Tasking Capability Descriptions for Existing IoT Products

**Figure 8:**
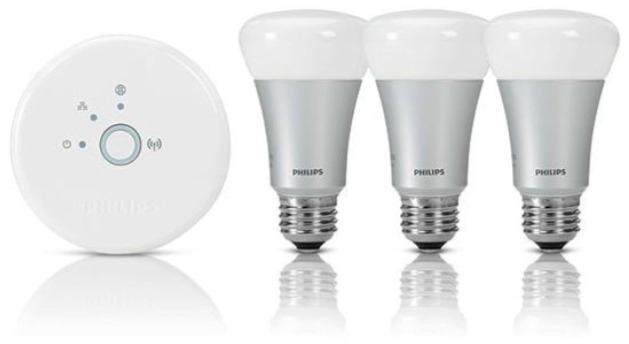
The Philips Hue.

As shown in Figure 8, the Philips Hue is a smart device that can be remotely controlled to change the colors, brightness, and saturation of light bulbs. While the Philips Hue is one of the popular IoT devices, users need to use their applications or follow their proprietary protocol to control the device. If our proposed tasking capability description can describe the service protocol to control Hue light bulbs, users can control them with the same protocol as other describable IoT devices.

**Figure 9:**
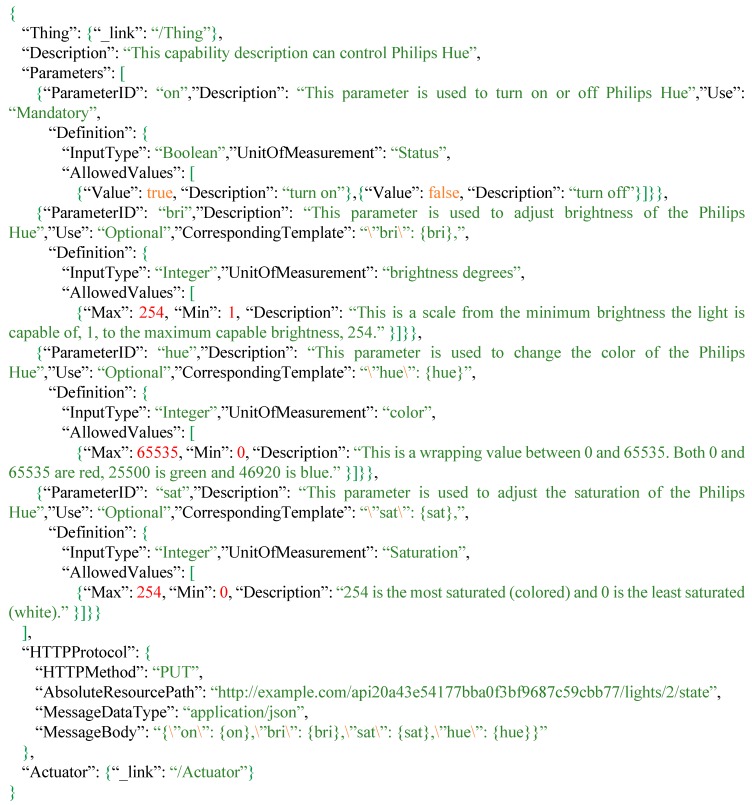
The tasking capability description of Philips Hue.

Therefore, based on the Application Programming Interface (API) of Hue (http://www.developers.meethue.com/), we create the Hue tasking capability description as shown in Figure 9. A *TaskingCapability* of Hue links to a *Thing*, which could be a lamp. Description shows users that this tasking capability can be used to control Hue. *Parameters* record the acceptable parameters and feasible values so that users can know how to control Hue through the Extended SensorThings API. As shown in Figure 9, “on” can be used to turn on or off the Hue, “bri” is for adjusting the brightness, “hue” helps users change the color of Hue and “sat” can be used to adjust the saturation of Hue. The AllowedValues can be represented with *Range* and/or *Value*.

In order to describe the communication protocols of different IoT devices, *HTTPProtocol* can record the HTTP protocol. According to Figure 9, Hue accepts the HTTP PUT requests. AbsoluteResourcePath records the web service entry to control this Philips Hue, and MessageBody records the template that will be used to compose device requests. As mentioned in Section 3.3, this research designs the keyword “{ParameterID}” as the placeholder, such as the {on}, {bri}, {sat}, and {hue}, which will be replaced with users’ input values. Finally, the *TaskingCapability links to an Actuator* that enables this capability.

**Figure 10:**
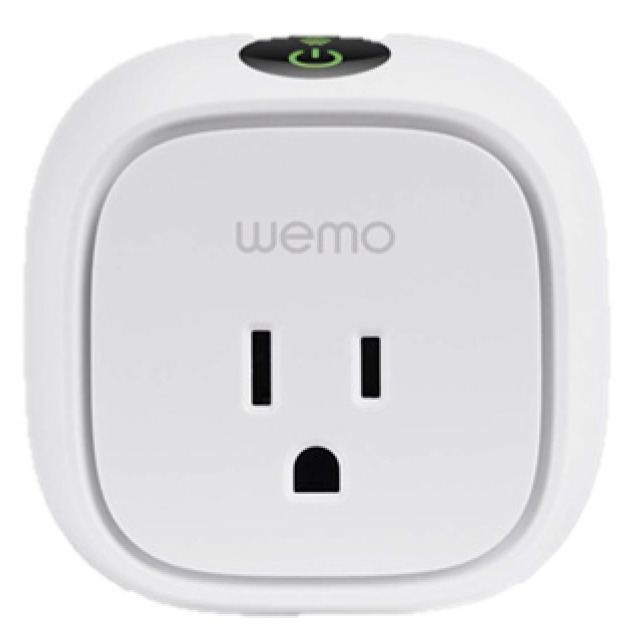
The Belkin WeMo InsightSwitch.

As shown in Figure 10, the Belkin WeMo InsightSwitch is a smart plug connecting to the Internet via WIFI. Users can connect some appliances like a dehumidifier and further remotely control this smart plug to turn on/off those connected appliances.

**Figure 11:**
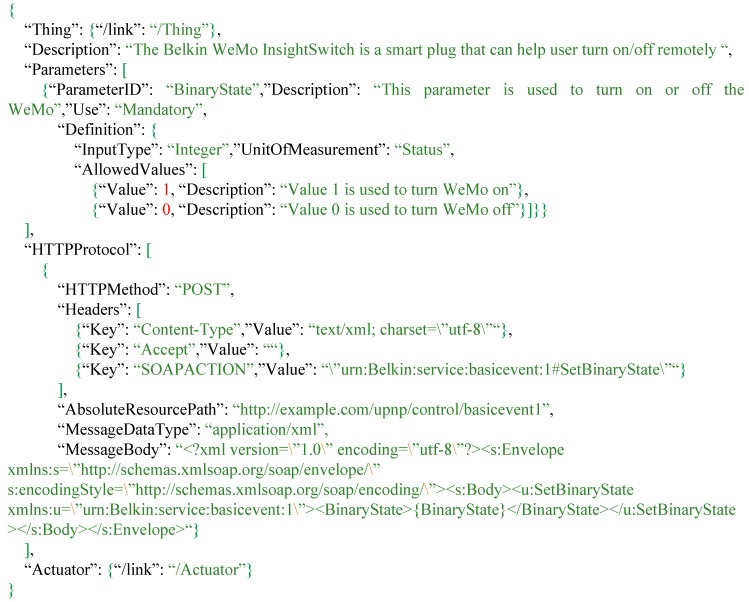
The tasking capability description of Belkin WeMo.

As shown in Figure 11, this description explains the Belkin WeMo InsightSwitch tasking capability. According to the WeMo InsightSwitch API, there is only one acceptable parameter called “BinaryState”, which allows users to turn on or off this WeMo smart plug. Figure 11 shows that value “1” and value “0” stand for “turn on” and “turn off”, respectively. From the *HTTPProtocol*, the Extended SensorThings API can understand the components for composing a device request. For example, the WeMo InsightSwitch requires some information in HTTP headers. In addition, the MessageBody stores the template of the XML document for device requests, where the {BinaryState} is the keyword placeholder for users’ input values.

**Figure 12:**
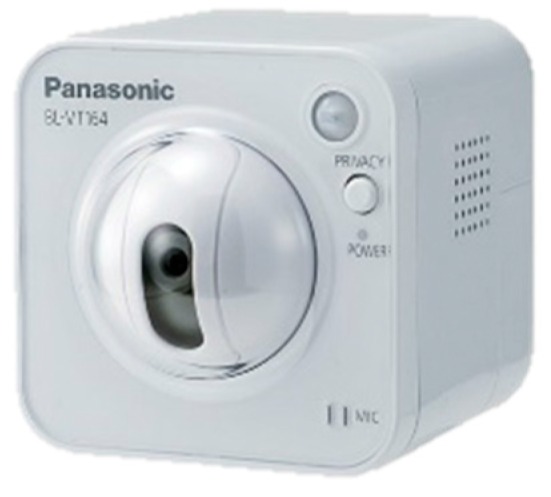
The Panasonic IP camera

**Figure 13:**
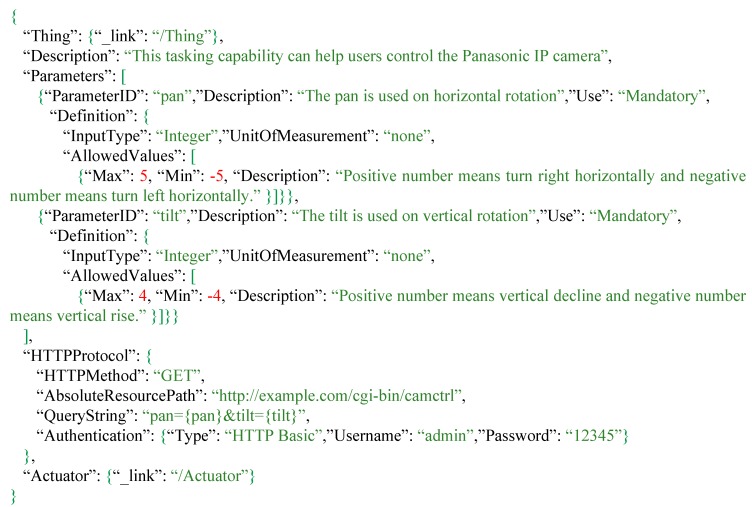
The tasking capability description of Panasonic IP camera.

The third IoT product we examined is the Panasonic IP camera as shown in Figure 12. IP cameras are common IoT devices, which allow users to remotely monitor features of interest in real time via the Internet. Therefore, the proposed solution should also support IP camera tasking capabilities. As shown in Figure 13, Description and Parameters provide information for users to understand how to task this IP camera. While the request template is also recorded in the *HTTPProtocol*, but different from the previous two IoT products, the request is an HTTP GET request, and the parameters are specified in query options (i.e., the {pan} and {tilt}).

In addition, the Panasonic IP camera supports authentication for privacy and security purposes. In order to automatically compose device requests, the authentication standard type, username and password are specified in the *HTTPProtocol* as well. This use case indicates that our proposed solution can record variable device protocols as the manufacturers of IoT devices wants.

### 4.2. The Task for Controlling IoT Products

According to these tasking capability descriptions, users can understand every detail of different tasking capabilities from different IoT devices and know how to use those acceptable parameters to create a Task entity to control these devices. Similar to other entities in SensorThings API, by creating tasking capability entities in an Extended SensorThings API, users can get a unique ID for each *TaskingCapability*. A user can use the *TaskingCapability* ID to specify which tasking capability of which devices in the *Task* entity.

**Figure 14:**
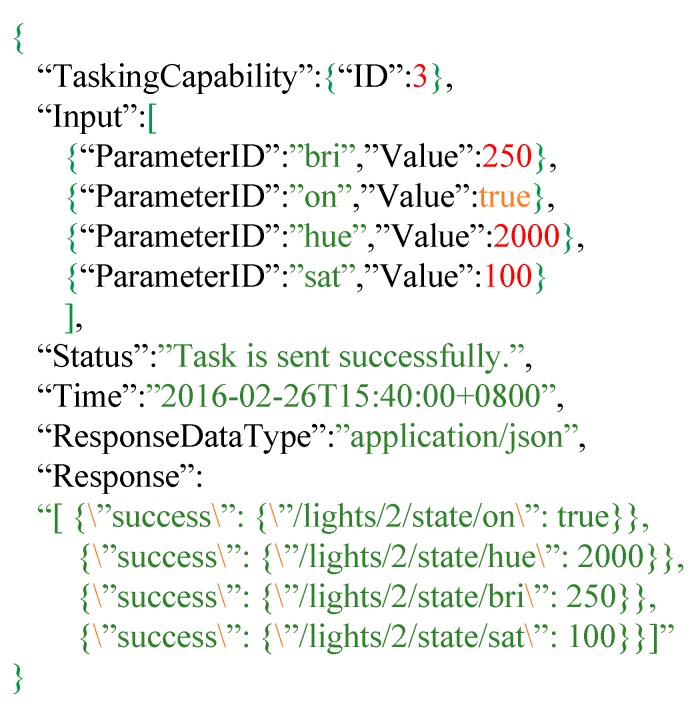
A *Task* entity example for controlling Philips Hue.

In order to control those IoT devices, users can send a *Task* to the Extended SensorThings API by setting the acceptable input values. Figure 14 shows an example of a *Task* entity for controlling a Philips Hue. As shown in Figure 14, *TaskingCapabilitiy* records those IDs for controlling the specific IoT devices. Input records the user’s command values. The example *task* sets “on” is *true* to turn on the Philips Hue, change the color to red by “hue”, set the high brightness to with “bri” and 250 and adjust the lower saturation by “sat”. The *Time* is the time that the user wants to control the IoT device. According to the example, at 26 February 2016 15:40, the Extended SensorThings API will send the device request to the IoT devices. *Status* describes the status of this Task, while the Task is sent successfully, status would tell us “Task is sent successfully”.

When an Extended SensorThings API receives the Task entity, the Extended SensorThings API will parse the task to retrieve the executing time, input parameters and values, and the corresponding *TaskingCapability* ID. Then the Extended SensorThings API will compose a device request by replacing keyword placeholders in the template with user’s input values. Finally, the Extended SensorThings API service sends the device request at the specified time. While IoT devices receive the device requests, some IoT devices would return responses to the service. In this case, optional properties ResponseDataType and Response record the data type of response and response itself, respectively. Users can retrieve this information from the *Task* entity.

### 4.3. Physical Mashup Applications

The previous section shows that the proposed tasking capability description can describe different IoT device protocols. In order to further demonstrate that the Extended SensorThings API can handle both IoT sensing and tasking capabilities, this study implements two physical mashup examples based on the Extended SensorThings API. To capture sensor observations, this study connects the open-sourced Arduino microcontroller with sensors and communication modules. According to the SensorThings API standard, Arduino sensor nodes can send observations of different environmental properties to an Extended SensorThings API service.

**Figure 15:**
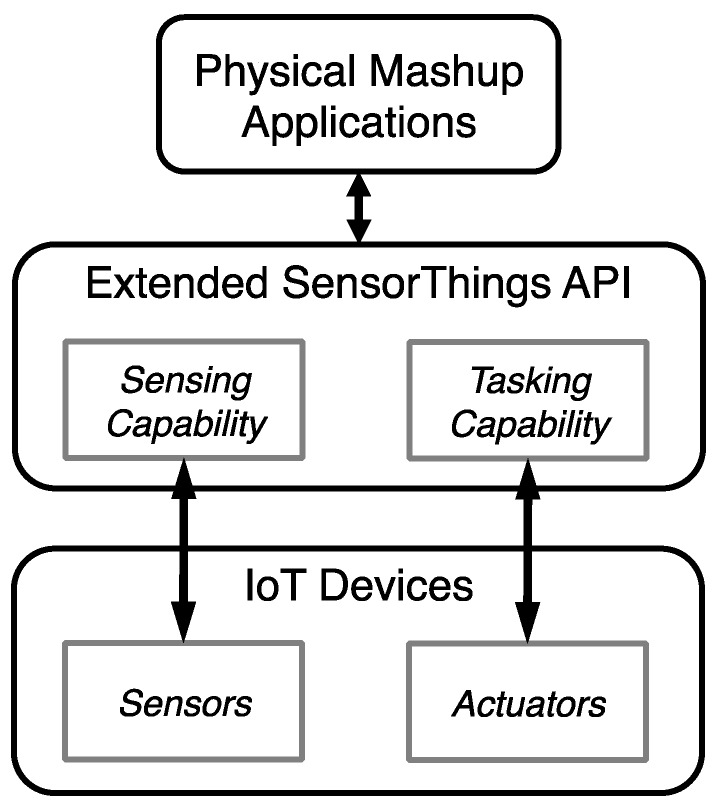
The high-level IoT architecture using the Extended SensorThings API.

In general, Figure 15 shows the high-level IoT architecture using the Extended SensorThings API. While IoT devices have sensors and actuators, the Extended SensorThings API has corresponding sensing and tasking capabilities to support them. Sensors can continuously upload observations to a service, and actuators and their tasking capabilities can be registered to a service. In this case, users or physical mashup applications can retrieve observations from different sensors and control different actuators through a coherent web service protocol. This paper uses two simple use cases as examples to demonstrate the contributions of the Extended SensorThings API.

#### 4.3.1. Use Case 1—Automatic Dehumidifier

People turn on dehumidifiers when they feel uncomfortable because of high relative humidity. In order to make the process more automatic and efficient, we can apply the Extended SensorThings API to collect relative humidity data from a sensor and automatically control a traditional dehumidifier via the WeMo Switch when the humidity exceeds a predefined threshold. Although there have been similar products integrating a humidity sensor in a dehumidifier, we simply want to demonstrate that without buying a more expensive product, similar functionality can be achieved by simply connecting sensing and tasking capabilities from different connected devices.

**Figure 16:**
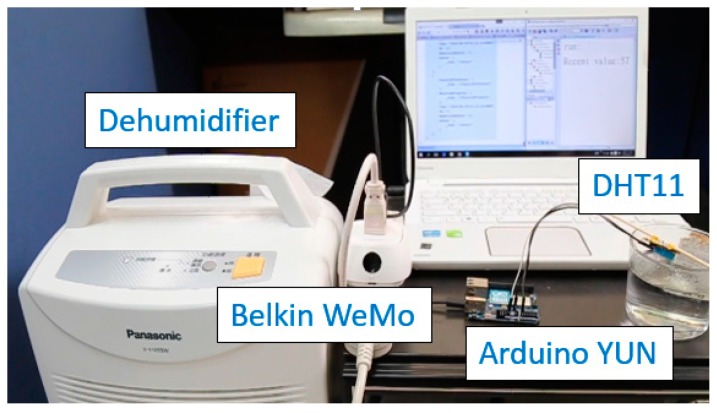
The prototype of an automatic dehumidifier.

Figure 16 shows the prototype of an automatic dehumidifier. For the sensing capability, this study uses the DHT11 as the humidity sensor to measure the relative humidity and connects the DHT11 with Arduino YUN to upload observations to an Extended SensorThings API service every 30 min. From the data model perspective, we assigned the “room” as a *Thing*, the DHT11 as the *Sensor*, and “relative air humidity” as the *ObservedProperty*. Users and applications can then retrieve the time-series *Observations* from the service.

Regarding the tasking capability, we connected a traditional dehumidifier to the WeMo Switch. While the integrated system is considered as another *Thing*, the WeMo Switch is the *Actuator*, and its *TaskingCapability* is similar to the example shown in Figure 11. By connecting the WeMo Switch with the dehumidifier, users can remotely turn on/off the machine.

After registering both the sensor node and dehumidifier to an Extended SensorThings API service, an application can first periodically retrieve humidity readings from the service. When the humidity exceeds the predefined maximum or minimum thresholds, the application will automatically turn on or off the dehumidifier by creating a corresponding *Task* entity in the Extended SensorThings API service.

#### 4.3.2. Use Case 2—A Smart Office Lighting System

**Figure 17:**
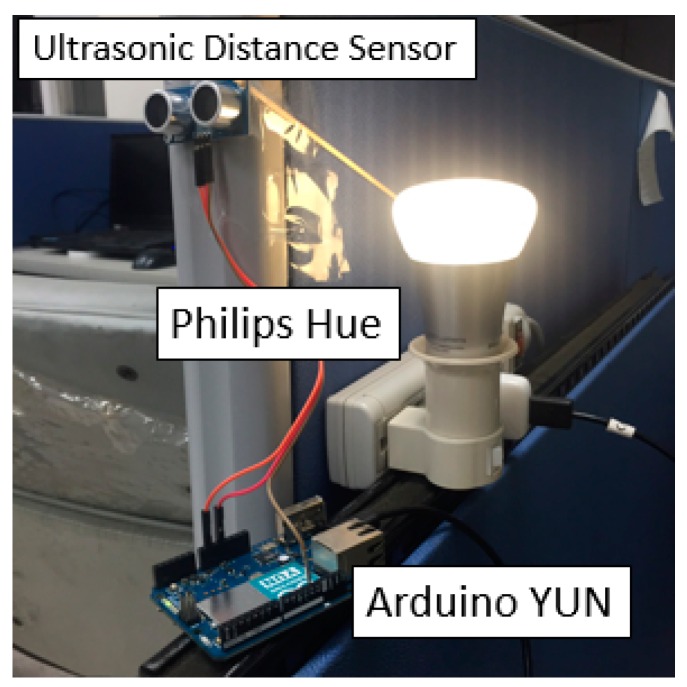
The prototype of a smart office light system.

In daily life, many office lighting systems consume unnecessary energy when no one is present, or people often forget to turn off the lights when they leave the office. To address this issue, this study implements a simple application that uses an ultrasonic distance sensor to monitor the distance changing in a hallway and automatically adjusts the Philips Hue light bulbs, as shown in Figure 17.

This study deploys an ultrasonic distance sensor on the wall and connects the sensor to an Arduino YUN. The sensor produces measurements at a high frequency (i.e., five readings per second). Once the reading changes (e.g., when someone passes by), the Arduino YUN will upload an *Observation* entity to an Extended SensorThings API automatically. Then, similar to the previous application, an application can periodically retrieve readings from the service and automatically turn on/off the Philips Hue light bulbs.

In general, this study modeled the “office” as a *Thing*, the “ultrasonic distance sensor” as the *Sensor*, and “distance” as the *ObservedProperty*. Then the Philips Hue was considered as another *Thing* and *Actuator*, whose *TaskingCapability* is similar to the Figure 9.

## 5. Conclusions and Future Work

This paper proposes an interoperable solution for realizing the IoT tasking capability. While large heterogeneities exist in the current IoT device communication protocols, to control different devices, users/applications need to use or implement various customized connectors. To address this issue, this research proposes a data model and JSON-based description of IoT tasking capability. While the tasking capability can be used to describe the functionalities and communication protocols of IoT devices, we design and implement a web service that can understand the tasking capability and translate users’ input tasks into device requests. In this case, users can control heterogeneous IoT devices via a coherent web service interface.

In addition, the proposed tasking capability was designed to integrate with the OGC SensorThings API to handle both IoT sensing and tasking capabilities in a single web service, which is called the Extended SensorThings API. To demonstrate the contribution of this study, we first applied the proposed tasking capability on three existing IoT products and successfully modeled their device protocols. Furthermore, this study implements two simple applications using the Extended SensorThings API. These applications retrieve sensor observations periodically from the service and send tasks to control different IoT devices according to the observations. Overall, we believe that the proposed solution could address the heterogeneity problem of IoT devices, and consequently realize the Internet of Things vision.

For future work, as this paper mainly focuses on the tasking capabilities served with HTTP, we will explore the feasibility of supporting different communication protocols such as Constrained Application Protocol (CoAP) and formerly MQ Telemetry Transport (MQTT). If those protocols can be described via the proposed tasking capability, the web service implementation could also be extended to support more possible IoT device protocols. Finally, as the proposed solution could be an extension to the OGC SensorThings API for handling the tasking capability, we plan to promote and introduce this solution to the OGC SensorThings API Standards Working Group.

## Figures and Tables

**Table 1:** Properties of the *TaskingCapability*.

Name	Definition	Data Type	Multiplicity and Use
Description	A human-readable description for the Tasking Capability	CharacterString	One (Mandatory)

**Table 2:** Properties of the *Actuator*.

Name	Definition	Data Type	Multiplicity and Use
Description	A human-readable description for the Actuator	CharacterString	One (Mandatory)
EncodingType	Data type of the metadata	ValueCode (e.g., for SensorML, application/xml, application/json)	One (Mandatory)
Metadata	A metadata of the actuator	Any	One (Mandatory)

**Table 3:** Properties of the *Parameter*.

Name	Definition	Data Type	Multiplicity and Use
ParameterID	The unique identification for an allowed parameter	CharacterString	One (Mandatory)
Description	A human-readable description for the Parameter	CharacterString	One (Mandatory)
Use	The necessity of the ParameterID	“Mandatory” or “Optional”	One (Mandatory)
CorrespondingTemplate	The corresponding portion of template in the *HTTPProtocol* of an optional ParameterID	CharacterString	Zero to one (Optional)

**Table 4:** Properties of the *Definition*.

Name	Definition	Data Type	Multiplicity and Use
InputType	The data type of the input value	“String”, “Double”, “Boolean”, “Integer”	One (Mandatory)
UnitOfMeasurement	Unit of measurement of the input value	JSON_Object	One (Mandatory)

**Table 5:** Properties of the *AllowedValues*.

Name	Definition	Data Type	Multiplicity and Use
Value	An acceptable value for the Parameter	Value	Zero to many (at least one Value or one Range is required)
Range	An acceptable value range for the Parameter	Range	Zero to many (at least one Value or one Range is required)

**Table 6:** Properties of the *Value*.

Name	Definition	Data Type	Multiplicity and Use
Value	An acceptable value for the Parameter	Any	One (Mandatory)
Description	A human-readable description of the Value	CharacterString	One (Mandatory)

**Table 7:** Properties of the *Range*.

Name	Definition	Data Type	Multiplicity and Use
Min	The minimum value of a range	Double or Integer	One (Mandatory)
Max	The maximum value of a range	Double or Integer	One (Mandatory)
Description	A human-readable description of the Range	CharacterString	One (Mandatory)

**Table 8:** Properties of the *HTTPProtocol*.

Name	Definition	Data Type	Multiplicity and Use
HTTPMethod	The HTTP method of the device HTTP request	HTTP Method	One (Mandatory)
AbsoluteResourcePath	Absolute resource path of the device HTTP request	URL	One (Mandatory)
MessageBodyDataType	The data type for the *MessageBody*, which can be used to verify the content in the *MessageBody*	ValueCode (e.g., for application/xml, application/json)	Zero to one (Optional)
MessageBody	The MessageBody of the device HTTP request	CharacterString	Zero to one (Optional)
QueryString	The QueryString of the device HTTP request	CharacterString	Zero to one (Optional)
Headers	The Headers of the device HTTP request	CharacterString	Zero to many (Optional)
Fragment	The Fragment of the device HTTP request	CharacterString	Zero to one (Optional)
Authentication	Authentication information for accessing the IoT device	Authentication	Zero to one (Optional)

**Table 9:** Properties of the *Authentication*.

Name	Definition	Data Type	MultIPLICITY and Use
Type	Type of HTTP authentication	Value code (e.g., for HTTP Basic, HTTP Digest)	One (Mandatory)
Account	User accounts for accessing IoT devices	CharacterString	One (Mandatory)
Password	Password for accessing IoT devices	CharacterString	One (Mandatory)

**Table 10:** Properties of the *Task*.

Name	Definition	Data Type	Multiplicity and Use
Time	Time for executing the task. If the Time is not specified, the request will be sent immediately.	ISO 8601 Time String	Zero to one (Optional)
Status	The task status automatically changed by the service	CharacterString	One (Mandatory)
ResponseDataType	The data type of the response from device	ValueCode (e.g., for application/xml, application/json)	Zero to one (Optional)
Response	The response message from device	Any	Zero to one (Optional)

**Table 11:** Properties of the *Input*.

Name	Definition	Data Type	Multiplicity and Use
ParameterID	The unique identification of the *Parameter*	CharacterString	One (Mandatory)
Value	User’s input value	Any (but limited by the *Parameter’s Definition* and *AllowedValue*)	One (Mandatory)
